# Efficacy of a psychotherapeutic group intervention for patients with Post-COVID-19 condition: a randomized controlled trial (PsyLoCo study)

**DOI:** 10.1186/s40359-026-05149-2

**Published:** 2026-07-23

**Authors:** Caroline Rometsch, Kimberly Colditz, Melanie Elgner, Christine Allwang, Stephan Zipfel, Florian Junne, Marius Binneböse

**Affiliations:** 1 Department of Psychosomatic Medicine and Psychotherapy, Medical University Hospital Tuebingen, Osianderstr.5, Tübingen, 72076 Germany; 2 German Center for Mental Health (DZPG), Partner Site Tübingen, Berlin, Germany; 3 Medical Faculty, University Clinic for Psychosomatic Medicine and Psychotherapy, University Medicine, Otto-Von-Guericke-University Magdeburg, Leipziger Str. 44, Magdeburg, 39120 Germany; 4 German Center for Mental Health (DZPG), Partner Site Halle-Jena-Magdeburg, Magdeburg, Germany; 5 Center for Behavioral Brain Sciences (CBBS), Magdeburg, Germany; 6 TUM School of Medicine and Health, Technical University of Munich, Ismaninger Str. 22, 81675 Munich, Germany

**Keywords:** SARS-CoV-2-infection, Post-COVID-19, Persistent physical symptoms, Group psychotherapy, Fatigue

## Abstract

**Background:**

Persistent physical symptoms such as fatigue following SARS-CoV-2 infection are common and often accompanied by cognitive complaints. Alongside biological mechanisms, psychosocial processes can contribute to symptom persistence. Psychotherapeutic interventions could be beneficial in symptom reduction. However, evidence of group-based psychotherapies tailored to Post-COVID-19 condition (PCS) remains limited.

**Methods:**

In this randomized waitlist-controlled crossover study in an outpatient psychosomatic setting, adults with prior SARS-CoV-2 infection and persisting functionally impairing symptoms were randomized to immediate group psychotherapeutic treatment (intervention group, IG) or treatment as usual with delayed group treatment (control/waitlist group, CG). The intervention comprised 10 bi-weekly manual-guided group sessions (50 min) covering psychoeducation, somatic symptom processing, emotional regulation, and social reintegration. Primary outcome was somatic symptom burden (SSS-8). Secondary outcomes were depressive symptoms (PHQ-9) and anxiety symptoms (GAD-7). Childhood maltreatment (CTS) was examined as a moderator. Efficacy in the randomized phase was tested using ANCOVA comparing post-treatment outcomes in the IG with post-waiting outcomes in the CG, adjusted for baseline severity. Three-month follow-up assessments were analyzed descriptively and exploratorily. Trial registration: https://osf.io/tdn38/overview on Feb 13, 2024 and German Clinical Trials Register DRKS00040598, registered on 12th June 2026. Retrospectively registered.

**Results:**

Forty-eight patients were included (IG *n* = 21; CG *n* = 27; mean age 53.3 years; 68.8% females). At baseline, somatic symptom burden was high (SSS-8 M = 15.21; *SD* = 5.62) with clinically relevant depressive symptoms (PHQ-9 M = 10.94, *SD* = 4.61) and moderate anxiety (GAD-7 M = 7.22; *SD* = 4.38). Fatigue-related physical exhaustion and cognitive complaints were the most prominent symptoms. No significant efficacy of the psychotherapeutic group intervention over TAU was observed for SSS-8 (β = − 2.20, SE = 1.25, 95% CI [− 4.77, 0.38], t(27) = − 1.75, *p* = .091), PHQ-9 (β = − 1.77, SE = 1.51, 95% CI [− 4.87, 1.33], t(26) = − 1.18, *p* = .251), or GAD-7 (β = − 0.81, SE = 1.47, 95% CI [− 3.84, 2.22], t(26) = − 0.55, *p* = .588) comparing post-treatment outcomes in the IG with post-waiting outcomes in the CG. Baseline symptom severity robustly predicted post-randomization outcomes during the randomized phase (*p* < .001). CTS was no moderator for the intervention effect.

**Conclusions:**

In this exploratory randomized controlled trial, the intervention did not show significant superiority over the control condition; findings should be interpreted cautiously given limited statistical power. Future psychotherapeutic group interventions for patients with PCS have to consider outcome measures beyond severity of persistent physical symptoms rather than evaluating global symptom scores. Group-based psychotherapeutic approaches might benefit from flexibility in adaptation for individual patients’ needs. Stratification by baseline severity and dominant symptom profiles (e.g., fatigue severity, trauma history) may improve sensitivity to clinically meaningful change.

**Trial registration:**

Trial registration: DRKS German Clinical Trials Register DRKS00040598, registered on 12th June 2026. Retrospectively registered.

## Introduction

Post-COVID-19 condition (PCS) refers to the presence of diverse persistent physical symptoms following SARS-CoV-2 infection, occurring beyond 12 weeks after symptom onset [9]. The pooled prevalence of PCS is estimated at 13.6% based on routine healthcare records [35].

Based on the current knowledge, PCS results from multisystem pathophysiological processes involving immune dysregulation, persistent inflammation [4], cerebral hypoperfusion, impaired pulmonary gas exchange [6], autonomic and neuropsychological impairment [31], while endothelial dysfunction, hypercoagulability, mast cell activation, and vascular autoimmunity show moderate to weak evidence [6]. Mechanisms of PCS further are currently understood to include perceptual and stress-related dysfunctions (e.g., autonomic/HPA-axis dysregulation, heightened interoceptive processing, maladaptive cognitions, behaviour amplifications) [15]. Childhood trauma has additionally been associated with increased depression, anxiety, and reduced health-related quality of life in patients with PCS [30]. In some patients, persistent symptom burden may overlap with diagnostic features of the somatic symptom disorder (SSD) according to DSM-5 [19], which encompass the presence of one or more distressing somatic symptoms accompanied by excessive and disproportionate health-related thoughts, feelings, or behaviours persisting for at least six months [24].

To date, pharmacological approaches for patients with PCS remain heterogeneous, with some evidence for symptom-specific improvements (e.g., sulodexide for endothelial dysfunction, chest pain; ivabradine for palpitations; vitamin D for olfactory and gustatory dysfunction; probiotics for fatigue [5], agomelatine [21]), however, results remain insufficient to support generalizable treatment recommendations [5]. In contrast, cognitive behavioural therapy (CBT) and combined physical and mental health rehabilitation seems to be effective for improving fatigue, quality of life, and overall recovery [36] in patients with PCS. Against this background, a structured, manualised, modular psychotherapeutic intervention specifically tailored to the psychosocial needs of patients with PCS for individual sessions was developed within the PsyLoCo pilot-study [1] comprising four core modules: (1) stabilization and psychoeducation (e.g., careful anamnesis, symptom validation, guideline-consistent information, relaxation techniques such as progressive muscle relaxation, and resource activation); (2) somatic symptom processing (e.g., development of a biopsychosocial illness model, symptom monitoring via diaries, and attention-shifting strategies to reduce hypervigilance); (3) mood and fatigue management (e.g., CBT- and Acceptance and Commitment Therapy (ACT)-compatible techniques such as mindfulness and cognitive defusion, values clarification, and energy management); and (4) social and vocational reintegration (e.g., individualized graded re-engagement plans, management of overexertion and perfectionism, and support for workplace communication) [12]. Evaluation of the efficacy of this psychotherapeutic intervention is currently ongoing.

Based on this psychotherapeutic manual, a group-based intervention has subsequently been developed. The present article examines the efficacy of this manual-guided group intervention for patients with PCS aiming at answering the following research questions:

1. To determine whether this pilot randomized controlled study applying a group intervention leads to a significant reduction in persistent physical and psychological symptom burden compared with treatment as usual (TAU).
2. To examine whether exposure to childhood maltreatment moderates therapeutic response.
3. To investigate whether baseline symptom severity predicts efficacy of symptom improvement over time.

## Methods

### Study design

**Fig. 1:**
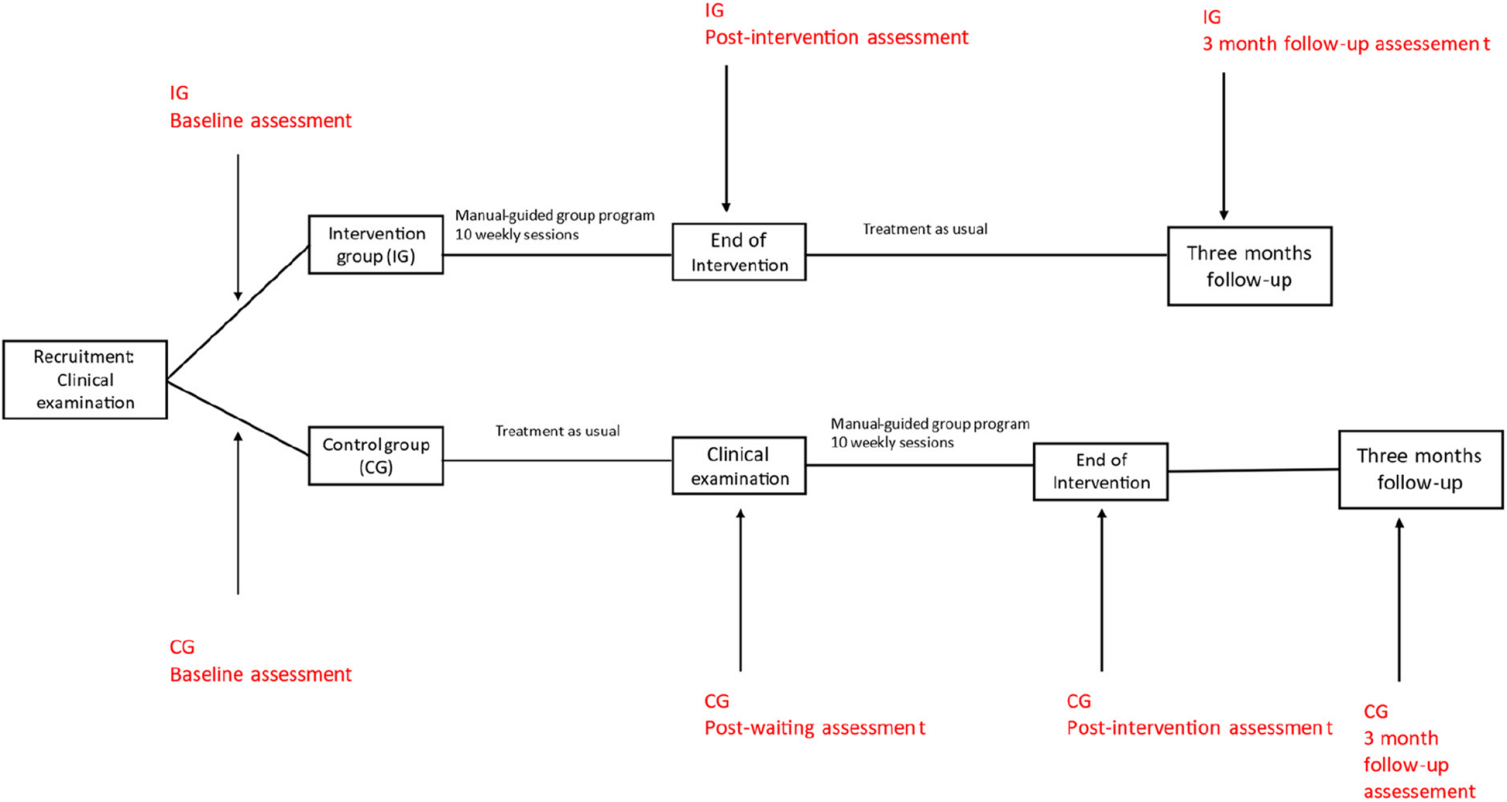
Study design and assessment schedule of the manual-guided group psychotherapy for patients with Post-COVID-19 condition. Note. IG = intervention group, CG = control group

This randomized controlled trial with a longitudinal design included two parallel conditions: (a) immediate treatment (intervention group; IG) or (b) delayed treatment after waiting time (control group; CG) with 1:1 ratio allocation. Both IG and CG underwent baseline assessment. The IG immediately received the manual-guided group intervention consisting of 10 bi-weekly psychotherapeutic sessions, followed by post-treatment assessment and a three-month follow-up assessment. After completion of the intervention, participants in the IG continued with TAU comprising routine medical care (e.g., general practitioner consultations, physiotherapy) without any structured psychotherapeutic interventions. Patients in the CG initially received TAU after baseline assessment and completed a post-waiting assessment prior to crossover treatment. Subsequently, the CG received the same manual-guided group psychotherapy followed by a post-treatment assessment after crossover and a 3 month follow-up assessment (see Fig. 1).

### Ethical considerations

Ethical approval was obtained from the Otto-von-Guericke-University of Magdeburg, Germany (ethical votum: 147/23). All patients received written and oral information about the study procedures, potential benefits and risks, confidentiality, rights to withdraw at any time without disadvantages, and provided written informed consent prior to assessment. The study was conducted in accordance with the principles of the Declaration of Helsinki [2]. Data were collected and pseudonymized, with the allocation list kept separately from outcome data and accessible only to authorized study personnel. The study protocol was preregistered on OSF [7, 10] as a bicentric trial in Magdeburg and Munich. Deviating from the preregistration, recruitment at the Munich site was not feasible, and the present analyses are therefore based on the Magdeburg sample only. Further preregistered outcomes (i.e., psychosocial and feasibility-related outcomes) will be published separately due to the extensive scope of the dataset. No outcome analyses reported here were selectively chosen post hoc. This is a pilot study that was designed as an exploratory randomized controlled trial. No formal a priori power calculation was performed.

### Sample description

The trial was conducted in an outpatient psychosomatic care setting of the University hospital Magdeburg, Germany. Eligible patients were adults (≥ 18 years) with (1) a reported prior SARS-CoV-2 infection (confirmed by antigen test) and (2) at least one persisting symptom with clinically relevant functional impairment. Exclusion criteria were severe mental disorders (e.g., acute psychosis, bipolar disorder, severe substance use disorder, acute suicidality), current psychotherapy, insufficient German.

### Recruitment procedure

The study procedure followed a two-step screening approach: (1) initial eligibility screening by telephone, and (2) clinical confirmation by physicians trained in psychosomatic medicine. Randomization was performed using a computer-generated randomization list. Each patient received a pseudonymized study identifier prior to allocation. Given the nature of a psychotherapeutic group intervention, patients and therapists were not blinded to treatment allocation; however, allocation was concealed until assignment, and analyses were conducted on pseudonymized data.

### Intervention: manual-guided psychotherapeutic group program

The intervention consisted of a structured, manual-guided group psychotherapy program delivered in 10 bi-weekly sessions of 50 min each. The program was based on a modular psychotherapeutic framework specifically developed for patients with PCS [7], addressing key psychosocial mechanisms implicated in persistent somatic symptoms. Core components included stabilization and psychoeducation, somatic symptom processing, mood and fatigue management, and social and vocational reintegration [1]. While the original manualized intervention was designed as a 12-session program, the present study applied a condensed 10-session group format while retaining the full thematic scope delivered in a bi-weekly rhythm. The intervention was embedded within a biopsychosocial, resource-oriented treatment model and conducted by two trained therapists (psychologists and/or physicians specialized in psychosomatic medicine). Patients randomized to the CG received TAU during the waiting period (no structured psychotherapy) and were offered the same group intervention following completion of the randomized phase. Harms were assessed non-systematically. Session attendance and missed sessions were documented throughout the intervention period. In addition, therapists closely adhered to the treatment manual to support treatment consistency and fidelity.

### Assessment instruments

1. Severity of persisting physical symptoms

The primary outcome was the severity of persisting physical symptoms assessed with the German version of the Somatic Symptom Scale–8 (SSS-8) [13], a brief self-report instrument to assess the severity of somatic symptoms. The SSS-8 has a structure reflecting major symptom clusters, including gastrointestinal, cardiopulmonary, pain, fatigue-related symptom domains [13]. The SSS-8 comprises 8 items rated on a 5-point Likert scale (0 = *“not at all”* to 4 = *“very much”*) referring to symptoms experienced within the last 7 days, yielding a sum score ranging from 0 to 32, with higher scores indicating greater somatic symptom burden [13]. For interpretability, categories are available: 0–3 (no to minimal), 4–7 (low), 8–11 (medium), 12–15 (high), and 16–32 (very high) somatic symptom burden [13].

In addition, patients completed a symptom checklist to capture the spectrum of PCS. Using standardized items (i.e., “*Which symptoms do you currently experience and how would you rate their severity*?”), patients rated the severity of olfactory and gustatory complaints, rapid physical exhaustion following exertion (e.g., climbing stairs), memory impairment, concentration difficulties. All symptoms were assessed on a 6-point ordinal scale ranging from 0 (“*not at all*”) to 5 (“*very severe*”).

2. Depressive, anxiety and trauma symptoms

Secondary outcomes included depressive, anxiety, and trauma-related symptoms. Depressive symptoms were measured using the Patient Health Questionnaire-9 (PHQ-9) [20], a brief self-report instrument that assesses the nine DSM-IV criteria for depressive disorders over the last 2 weeks [20]. The PHQ-9 comprises 9 items rated on a 4-point Likert scale (0 = *“not at all”* to 3 = *“nearly every day”*), yielding a sum score ranging from 0 to 27, with higher scores indicating greater depressive symptom severity and a cutoff of ≥ 10 indicating relevant depressive symptom burden. For interpretability, severity thresholds are 5 (“*mild*”), 10 (“*moderate*”), 15 (“*moderately severe*”), and 20 (“*severe*”) depressive symptoms [20]. Anxiety symptoms were assessed using the Generalized Anxiety Disorder scale-7 (GAD-7) [29], a brief self-report instrument comprising 7 items rated on a 4-point Likert scale (0 = “*not at all*” to 3 = “*nearly every day*”) referring to symptoms experienced within the last 2 weeks, yielding a sum score ranging from 0 to 21, with higher scores indicating greater anxiety symptom severity and a cutoff of ≥ 10 as the threshold for detecting probable Generalized Anxiety Disorder [20]. For interpretability, recommended severity thresholds are 5 (“*mild*”), 10 (“*moderate*”), and 15 (“*severe*”) anxiety symptoms [20]. Retrospective exposure to childhood maltreatment was assessed with the Childhood Trauma Screener (CTS) [14], an ultra-brief self-report screening instrument derived from the Childhood Trauma Questionnaire [3] to efficiently capture core dimensions of childhood abuse and neglect. The CTS comprises five items belonging to emotional neglect, physical neglect, physical abuse, emotional abuse, sexual abuse (e.g., “*When I was growing up, I felt loved*”; “*People in my family hit me so hard that it left me with bruises or marks*”) rated on a 5-point Likert scale (1 = “*never true*” to 5 = “*very often true*”). A total score can be computed with a range from 5 to 25, with higher scores indicating greater exposure to childhood maltreatment.

### Statistical analysis

Analyses were performed in RStudio (version 4.5.1) [33]. Assessment instruments and symptoms of PCS were analyzed computing total scores and standard deviations if a minimum number of non-missing items was available: SSS-8 (≥ 7/8 items), PHQ-9 (≥ 8/9 items), GAD-7 (≥ 6/7 items), PCS symptom checklist (≥ 4/5 items), and CTS (≥ 4/5 items). Baseline characteristics are reported descriptively for the total sample and by randomized group (means [*SD*] or counts [%]); standardized mean differences (Cohen’s d) are provided where applicable. Age was compared using Welch’s two-sample t-test. Cohen’s d was calculated using the pooled *SD*. Categorical sociodemographic variables (sex, employment status, sick leave, education, income, country of birth) were tested using Pearson’s chi-square test; Fisher’s exact test was applied when expected counts were < 5. Efficacy of the group psychotherapeutic intervention was tested using analysis of covariance (ANCOVA without interaction) implemented via linear regression models. For the primary randomized efficacy analyses, post-randomization outcomes were defined as post-treatment outcomes in the IG and post-waiting outcomes in the CG. All analyses were adjusted for baseline symptom severity. Model estimates are reported as regression coefficients with standard errors, 95% confidence intervals, t-values, degrees of freedom, and *p*-values; the group coefficient represents the adjusted between-group difference during the randomized phase comparing post-treatment outcomes in the IG with post-waiting outcomes in the CG. Exploratory within-subject changes at follow-up after treatment completion were analyzed using paired t-tests. Childhood trauma total score was mean-centered for moderation models on an exploratory basis. To examine whether childhood trauma moderated treatment effects, interaction models were fitted by adding mean-centered scores of CTS and the group × mean-centered scores of CTS interaction to the ANCOVA specifications for each outcome. Predictors of symptom change were examined using linear regression with symptom change as the dependent variable and baseline severity, mean-centered scores of CTS, and group as predictors. All tests were two-sided with an alpha level of *p* = 0.05 with Bonferroni correction (α-Bonferroni *p* = 0.0167). All regression analyses were conducted using a complete-case approach (listwise deletion) without imputation, i.e., only patients with complete data on the respective variables were included. Consequently, the number of patients contributing to each analysis varied depending on outcome availability at the respective assessment time points. Given the exploratory pilot character and small sample size, no multiple imputation procedure was applied.

## Results

### Sample characteristics and sociodemographic data

**Fig. 2:**
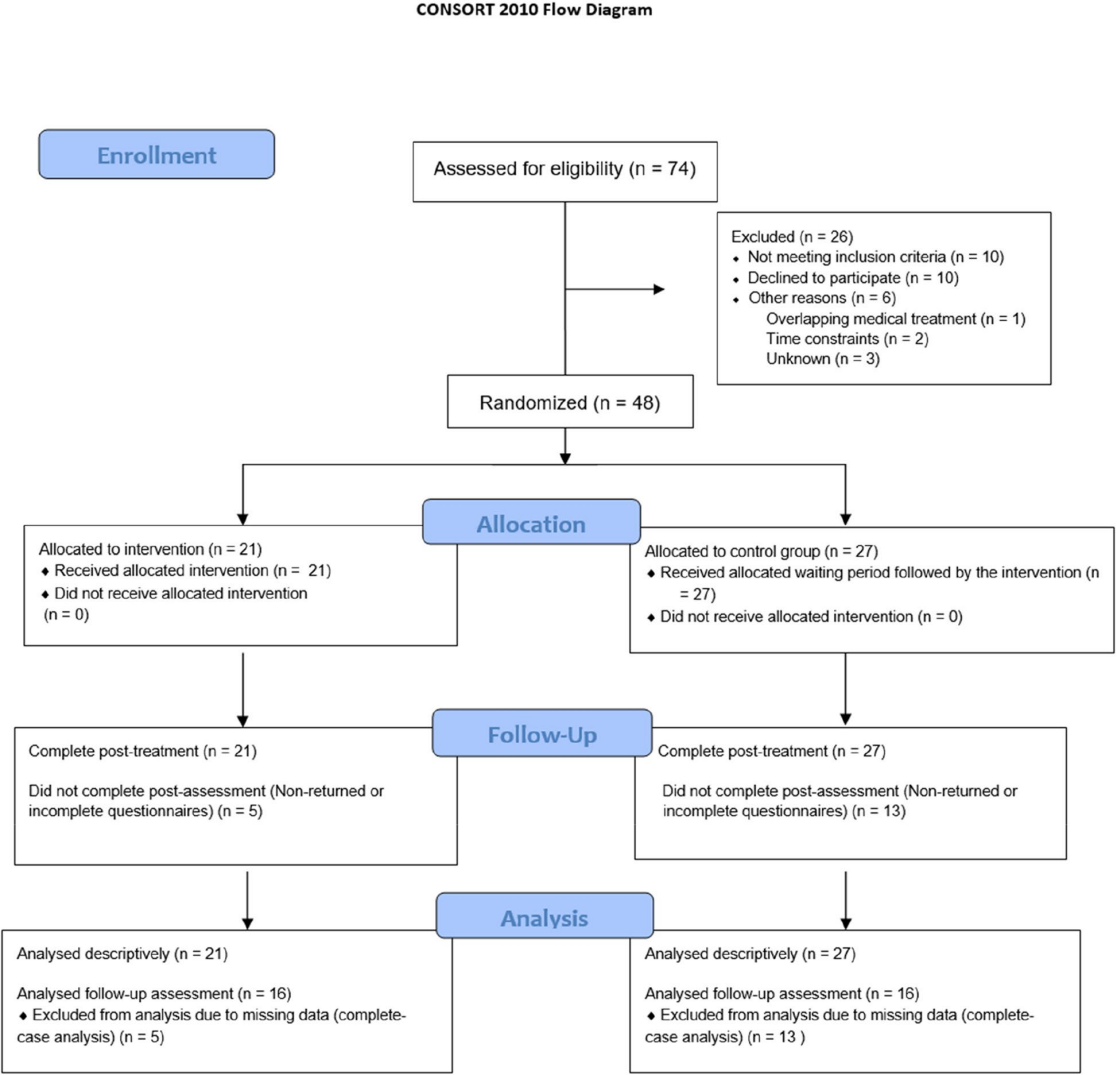
Flow chart of the recruitment, allocation, and dropout of patients with Post-COVID-19 condition (*N* = 48)

The number of patients contributing to each analysis varied depending on outcome availability and the crossover assessment schedule. Missing data primarily resulted from non-completion of follow-up questionnaires and discontinuation of participation during the intervention or waiting period. Baseline data were available for all randomized patients (total *N* = 48; IG: *n* = 21; CG: *n* = 27). Follow-up assessments after treatment in the IG included 16 patients for SSS-8, PHQ-9, and GAD-7. At post-randomization, data were available for the CG at the post-waiting assessment (before crossover) for 14 patients for SSS-8 and 13 patients for PHQ-9 and GAD-7. Follow-up assessments after crossover treatment in the CG included 16 patients for SSS-8 and GAD-7 and 16 patients for PHQ-9. Accordingly, complete-case ANCOVA models included fewer patients than initially randomized (SSS-8: *n* = 29; PHQ-9: n = 28; GAD-7: *n* = 28) (see Fig. 2).

The mean age of the total sample was 53.3 years (*SD* = 11.9; range 24–77). Patients of the IG were younger compared to the CG (51.7 years, *SD* = 12.0 vs. 54.6 years, *SD* = 11.9) although this difference was not statistically significant (*t*(43.04) = − 0.83, *p* = 0.411; Cohen’s *d* = − 0.24). The majority of patients were females (68.8% overall), with comparable sex distributions between groups (*χ*^2^(1) = 0.08, *p* = 0.784). No significant between-group differences were observed for employment status, sick leave at baseline, educational attainment, monthly income (see Table 1).

**Table 1:** Baseline sociodemographic and clinical characteristics of the total sample and separately for the intervention and control groups for psychotherapeutic group intervention for Post-COVID-19 condition

Characteristic	Total sample (*N* = 48)	Intervention group (*n* = 21)	Waitlist group (*n* = 27)
Age (years)	53.3 (*SD* = 11.9)	51.7 (*SD* = 12.0)	54.6 (*SD* = 11.9)
Sex, Females (n, %)	33 (68.8%)	14 (66.7%)	19 (70.4%)
Employment status, n (%)			
Employed (full- or part-time)	28 (58.3%)	11 (52.4%)	17 (63.0%)
Not employed	20 (41.7%)	10 (47.6%)	10 (37.0%)
Sick leave at baseline, n (%)			
Yes	5/10 (50.0%)	2/5 (50.0%)	3/5 (50.0%)
Highest school education, n (%)			
Secondary school	8 (16.7%)	5 (23.8%)	3 (11.1%)
Polytechnic school	19 (39.6%)	9 (42.9%)	10 (37.0%)
Higher education entrance qualification	21 (43.7%)	7 (33.3%)	14 (51.9%)
Monthly household income, n (%)			
< €1,250	4 (8.3%)	2 (9.5%)	2 (7.4%)
€1,250–1,750	4 (8.3%)	2 (9.5%)	2 (7.4%)
€1,750–2,250	12 (25.0%)	6 (28.6%)	6 (22.2%)
€2,250–3,000	4 (8.3%)	3 (14.3%)	1 (3.7%)
€3,000–4,000	13 (27.1%)	5 (23.8%)	8 (29.6%)
≥ €4,000	5 (10.4%)	2 (9.6%)	3 (11.1%)
N/A	6 (12.5%)	1 (4.8%)	5 (18.5%)
Country of birth, n (%)			
Germany	48 (100%)	21 (100%)	27 (100%)

### Primary outcomes: somatic complaints over the long-term course

**Fig. 3:**
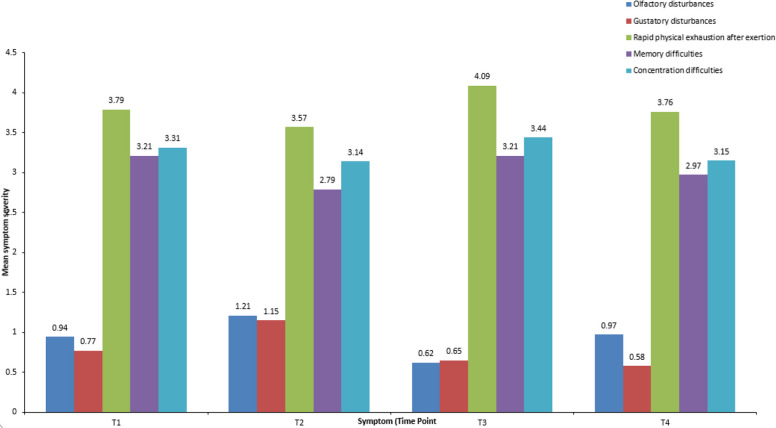
Mean severity scores (SD) for individual symptoms of Post-COVID-19 condition across all assessment time points for the total sample (*N* = 48) and stratified by intervention and control group

At baseline, mean SSS-8 scores were 15.21 (*SD* = 5.62). During the randomized phase, mean SSS-8 scores at assessment after completed intervention in the IG were 15.63 (SD = 6.38), whereas mean SSS-8 scores at post-waiting assessment before intervention in the CG were 14.21 (SD = 4.82). During the subsequent crossover phase, mean SSS-8 scores in the CG were 16.17 (SD = 4.69) after completed intervention and decreased to 14.59 (SD = 4.67) at follow-up assessment after completed intervention. In the IG, mean SSS-8 scores decreased to 13.38 (SD = 7.16) at follow-up assessment. Detailed descriptive results for each group and assessment time point are presented in Table 2. Severity of PCS assessed via the symptom checklist was highest for fatigue-related symptoms (e.g., rapid physical exhaustion after exertion (*M* = 3.79, *SD* = 1.18), concentration difficulties (*M* = 3.31, *SD* = 1.17), and memory difficulties (*M* = 3.21, *SD* = 1.30), whereas olfactory (*M* = 0.94, *SD* = 1.33) and gustatory disturbances (*M* = 0.77, *SD* = 1.15) were reported with lower severity at baseline. Across follow-up assessments, fatigue-related symptoms (e.g., rapid physical exhaustion) remained consistently elevated over time, both at IG follow-up assessment (*M* = 3.76, *SD* = 1.15) and at CG follow-up assessment after crossover treatment (M = 3.76, SD = 1.15). Similarly, concentration difficulties remained elevated at IG follow-up assessment (M = 3.44, SD = 1.13) and at CG follow-up assessment after crossover treatment (M = 3.15, SD = 1.25) (see Fig. 3). No baseline differences were detected between the IG and CG (see Table 2).

**Table 2:** Primary and secondary outcomes across assessment time points

Instrument	Time point	Total sample Mean (SD), n	Intervention group Mean (SD), n	Control group Mean (SD), n
Somatic symptom burden (SSS-8)	Baseline assessment	15.21 (5.62), 48	14.86 (5.82), 21	15.48 (5.56), 27
	Post -waiting assessment before intervention	-	-	14.21 (4.82), 14
	Assessment after completed intervention	15.92 (5.47), 34	15.63 (6.38), 16	16.17 (4.69), 18
	Three-month follow-up assessment after completed intervention	13.96 (6.02), 31	13.38 (7.16), 16	14.59 (4.67), 15
Depressive symptoms (PHQ-9)	Baseline assessment	10.94 (4.61), 48	10.38 (5.16), 21	11.37 (4.19), 27
	Post -waiting assessment before intervention	-	-	10.23 (4.59), 13
	Assessment after completed intervention	10.70 (5.12), 33	10.50 (6.58), 16	10.89 (3.42), 17
	Three-month follow-up assessment after completed intervention	9.74 (5.26), 33	9.56 (6.19), 16	9.90 (4.40), 17
Anxiety symptoms (GAD-7)	Baseline assessment	7.22 (4.38), 48	6.90 (3.86), 21	7.47 (4.80), 27
	Post -waiting assessment before intervention	-	-	7.54 (4.89), 13
	Assessment after completed intervention	7.41 (4.45), 34	7.69 (5.33), 16	7.16 (3.65), 18
	Three-month follow-up assessment after completed intervention	5.82 (4.15), 32	5.71 (4.63), 16	5.94 (3.77), 16
Childhood Trauma Scale (CTS)	Baseline assessment	11.95 (1.41), 48	11.85 (1.61), 21	12.04 (1.26), 27

### Secondary outcomes: depressive, anxiety, and trauma-related symptoms

At baseline, comorbid clinically relevant depressive (PHQ-9: *M* = 10.9, *SD* = 4.61) and anxiety symptoms (GAD-7: *M* = 7.22, *SD* = 4.38) were found. Exposure to childhood trauma was rather low across the sample (CTS: *M* = 12.0, *SD* = 1.41), with similar levels observed in the IG (*M* = 11.8, *SD* = 1.61) and CG (*M* = 12.0, *SD* = 1.26). No baseline differences were detected between the IG and CG. Across follow-up assessments, depressive and anxiety symptoms showed a tendency toward lower mean levels (see Table 2).

### Efficacy of the manual-guided group psychotherapy

#### Somatic symptom burden

Descriptively, the IG showed a slight increase in SSS-8 scores from baseline to post-treatment, whereas the CG showed a modest decrease during the waiting period from baseline to post-waiting assessment before intervention. No statistically significant group difference was observed during the randomized phase comparing assessment after completed intervention in the IG with post-waiting assessment before intervention in the CG (β = − 2.20, SE = 1.25, 95% CI [− 4.77, 0.38], t(27) = − 1.75, *p* = 0.091; p_adj = 0.273, α_Bonferroni = 0.0167). Baseline somatic symptom burden was a strong predictor of post-randomization SSS-8 scores during the randomized phase (β = 0.82, SE = 0.11, 95% CI [0.59, 1.06], t(27) = 7.17, *p* < 0.001).

#### Depressive symptoms

ANCOVA revealed no significant group difference in post-randomization depressive symptoms during the randomized phase comparing assessment after completed intervention in the IG with post-waiting assessment before intervention in the CG (β = − 1.77, SE = 1.51, 95% CI [− 4.87, 1.33], t(26) = − 1.18, *p* = 0.251; p_adj = 0.753, α_Bonferroni = 0.0167). Baseline depressive symptom severity was strongly associated with post-randomization PHQ-9 scores during the randomized phase (β = 0.87, SE = 0.16, 95% CI [0.55, 1.19], t(26) = 5.58, *p* < 0.001).

#### Anxiety symptoms

Similarly, no significant group difference was observed for anxiety symptoms during the randomized phase comparing assessment after completed intervention in the IG with post-waiting assessment before intervention in the CG (β = − 0.81, SE = 1.47, 95% CI [− 3.84, 2.22], t(26) = − 0.55, *p* = 0.588; p_adj = 1.000, α_Bonferroni = 0.0167). Baseline GAD-7 scores significantly predicted post-randomization anxiety levels during the randomized phase (β = 0.98, SE = 0.22, 95% CI [0.53, 1.43], t(26) = 4.50, *p* < 0.001).

#### Moderation by childhood maltreatment

No significant main effects of childhood trauma and no significant group × CTS interaction effects were observed for somatic symptom burden (SSS-8), depressive symptoms (PHQ-9), anxiety symptoms (GAD-7) when predicting post-randomization outcomes adjusted for baseline symptom severity (SSS-8: β = 0.12, SE = 1.45, 95% CI [− 2.86, 3.11], t(25) = 0.09, *p* = 0.932; PHQ-9: β = − 1.08, SE = 1.80, 95% CI [− 4.79, 2.64], t(24) = − 0.60, *p* = 0.556; GAD-7: β = − 0.22, SE = 1.88, 95% CI [− 4.11, 3.66], t(24) = − 0.12, *p* = 0.907) (all interaction *p* values > 0.55) (see Table 3).

**Table 3:** Moderation of treatment effects by childhood maltreatment for the manual-guided group psychotherapy for patients with Post-COVID-19 condition

Outcome	Predictor	B (SE)	95% CI	t	*p*
Somatic symptom burden (SSS-8)	CTS	0.15 (0.79)	[−1.47, 1.78]	0.20	.847
	Group (CG vs. IG)	− 2.33 (1.38)	[−5.16, 0.50]	− 1.69	.103
	Group × CTS	0.12 (1.45)	[− 2.86, 3.11]	0.09	.932
	Baseline SSS-8	0.83 (0.12)	[0.58, 1.08]	6.79	<.001*
Depressive symptoms (PHQ-9)	CTS	0.83 (0.90)	[−1.04, 2.69]	0.92	.369
	Group (CG vs. IG)	− 2.02 (1.68)	[−5.49, 1.45]	− 1.20	.241
	Group × CTS	− 1.08 (1.80)	[− 4.79, 2.64]	− 0.60	.556
	Baseline PHQ-9	0.90 (0.16)	[0.57, 1.24]	5.53	<.001*
Anxiety symptoms (GAD-7)	CTS	− 0.12 (0.93)	[−2.04, 1.80]	− 0.13	.899
	Group (CG vs. IG)	− 0.65 (1.66)	[−4.07, 2.78]	− 0.39	.700
	Group × CTS	− 0.22 (1.88)	[− 4.11, 3.66]	− 0.12	.907
	Baseline GAD-7	0.98 (0.24)	[0.48, 1.48]	4.07	<.001*

*SSS-8* Somatic Symptom Scale-8, *PHQ-9* Patient Health Questionnaire-9, *GAD-7* Generalized Anxiety Disorder-7, *CTS* Childhood Trauma Screener, *IG* intervention group, *CG* control group, *B* unstandardized regression coefficient, *SE* standard error, *CI* confidence interval

^*^significant on a *p* <.001-level

## Discussion

This pilot randomized controlled trial investigated a manual-guided group psychotherapy for patients suffering from PCS resulting in: 1. Cognitive and fatigue-related symptoms represented the most prominent and persistent symptom domains before and after intervention 2. No statistically significant efficacy of the immediate group intervention over the waiting control condition was observed, across primary and secondary outcomes 3. Baseline symptom severity consistently emerged as the strongest predictor of post-randomization outcomes. 4. Exposure to childhood maltreatment did not moderate efficacy of intervention.

Fatigue is the most prevalent persistent symptom after SARS-CoV-2 infection, affecting approximately 41% of survivors and is associated with female sex, older age, impaired physical functioning, breathlessness, psychological distress [23], while severe acute illness, multiple comorbidities, and premorbid depression or anxiety further increase the risk of Post-COVID-19 fatigue [18]. Cognitive symptoms include commonly memory and attention/executive problems (“brain fog”) [22]. Among available treatment approaches, psychotherapeutic interventions show the most consistent non-pharmacological benefits for post-viral fatigue, with group-based cognitive-behavioral and self-management/pacing interventions yielding the most reliable symptom reductions [11] along with physical approaches (e.g. exercise training). Additionally, multimodal programs integrating CBT-based psychotherapy are applied for patients with PCS, showing good feasibility, acceptability, and safety [26]. In this context, the substantial symptomatic and mechanistic overlap between PCS and myalgic encephalomyelitis/chronic fatigue syndrome, particularly regarding fatigue and cognitive impairment, provides a rationale for cognitive-behavioral approaches, for which moderate evidence indicates reductions in fatigue and improvements in cognitive function [32]. CBT-based approaches may be clinically relevant, particularly considering elevated prevalences of depression and anxiety in patients with PCS [27] predicting significantly symptom persistence and impairment (key modifiable targets for multidisciplinary treatment approaches) [8], overall shown when embedding CBT elements in broader rehabilitation or neurorehabilitation settings for improved subjective fatigue and disease coping [16].

However, the literature on group psychotherapeutic interventions tailored for PCS is limited. A low-threshold, outpatient group program consisting of eight weekly 90-min sessions, delivered by a psychotherapist and an internal medicine physician, followed a standardized session structure (structured check-in, brief psychoeducational input with visual aids, guided group discussion focusing on coping strategies, closing relaxation/imagination exercises), targeted fatigue and post-exertional malaise, stress intolerance, resource-oriented symptom management, and was complemented by medical question-and-answer sessions, physiotherapeutic components (breathing techniques and graded activation), optional social-work consultations, and a post-intervention booster session [37]. This intervention was shown to be feasible and well accepted for patients with PCS, with particular perceived benefit in peer exchange and validation of illness experience [37]. In an internet-delivered format, a manualized supportive group psychotherapy was delivered three times within one week (1–2 h per session) with a focus on supportive–expressive exchange, psychoeducation on PCS, normalization and validation of distress, coping strategies for fatigue and psychosomatic symptoms, and guided group discussion [28]. The intervention produced short-term reductions in self-reported psychological and somatic distress [28]. A non-randomized controlled trial applied an 8-week weekly group intervention combining group psychotherapy (CBT-based psychoeducation and coping) with computer-assisted cognitive training and revealed improvements in cognitive performance and depressive symptoms [17].

In contrast to the original manual developed for patients with PCS by Allwang and colleagues [1], this guided group psychotherapeutic approach differed in several design-relevant aspects that are likely to influence both mechanisms of change and outcomes. First, the intervention was shortened to 10 sessions, whereas the original concept was designed as a more comprehensive, modular psychosomatic program with 12 sessions and a stronger emphasis on individualized formulation. Second, the present adaptation necessarily reduced personalization intensity, as the fixed group sequence allows less flexibility for symptom-specific dose adjustment. Third, the delivery of sessions every two weeks increases the time between each session, which can attenuate behavioural learning cycles (e.g., graded activity, exposure to symptom-related cues, updating illness beliefs). However, in contrast to the original manual by Allwang et al. [1], the present adaptation deliberately retained core advantages of group-based interventions demonstrated in the literature (e.g., peer exchange, normalization, shared meaning-making), while extending the overall treatment duration through a bi-weekly delivery over a longer time frame, thereby allowing more sustained behavioural change processes, consolidation of coping strategies, gradual symptom-related learning within a psychosomatic framework that comprehensively acknowledges the multifaceted nature of PCS.

Against this background, the hypothesis for non-significant efficacy over TAU and CG on global symptom scales (e.g., somatic symptom burden, depressive symptoms, anxiety) may reflect a possible outcome–mechanism mismatch rather than evidence that the intervention is broadly ineffective although this interpretation remains exploratory due to small sample size and the absence of direct assessment of psychotherapeutic process variables. Further investigations are needed to manifest these results. Evidence from group psychotherapy research demonstrates that group-based interventions are generally effective and comparable to individual psychotherapy across a wide range of mental disorders, with treatment effects depending not only on dosage and session frequency but also on process-related factors such as group cohesion, therapeutic alliance, and feedback mechanisms [25]. Importantly, in complex and heterogeneous conditions such as PCS, psychotherapeutic effects may initially manifest in domains such as coping strategies, illness-related cognitions, self-efficacy, functional participation, quality of life, whereas changes in global symptom severity may emerge more slowly or indirectly [26].

Finally, childhood trauma was shown to be common among patients with PCS [34] and was associated with significantly higher rates of depression, anxiety, reduced health-related quality of life compared to healthy controls [30]. Recent trials support the integration of trauma-informed approaches into psychotherapeutic interventions for patients with PCS, suggesting that consideration of trauma-related factors may enhance treatment relevance and efficacy [30]. The present analyses on childhood trauma in patients with PCS should be interpreted cautiously due to the statistical challenges. Therefore, the absence of significant moderation effects cannot be interpreted as evidence that childhood trauma is unrelated to treatment response in patients with PCS. Furthermore, the present intervention did not include a dedicated trauma-focused module, and future adaptations may benefit from systematically integrating trauma-informed components to address the high prevalence of childhood adversity as well as modules particularly for posttraumatic stress disorders and its potential impact on symptom burden and treatment response in patients with PCS.

### Limitations

The sample size was modest, which may have limited power to detect small effects and moderation (e.g., childhood maltreatment), especially in a clinically heterogeneous population of patients with PCS. CTS scores showed relatively limited variability within the present sample with its limited sample size the statistical power for interaction effects was low. No a-priori power calculation was performed, which limits the interpretability of non-significant findings. Regression analyses were conducted using a complete-case approach, thus, only patients with complete data were included in the analyses. Consequently, attrition bias may occur during the randomized phase as missing data primarily resulted from non-completion of follow-up questionnaires or discontinuation of participation in the group intervention. Because analyses were based on complete cases for the respective outcome variables, the effective sample size differed between models. This may have reduced statistical power and limited the ability to detect small intervention effects. In addition, post-randomization dropout may have reduced the balance between groups initially achieved through randomization. In particular, patients with higher symptom burden, lower treatment adherence, or lower psychosocial functioning may have been underrepresented in later assessments, potentially biasing effect estimates and limiting the generalizability of findings. Outcomes were based on self-report and follow-up was limited to three months, which may not capture slower changes in functioning, coping, quality of life, that are central targets of psychosomatic psychotherapy. Although session attendance and missed sessions were documented throughout the intervention, no formal fidelity ratings or statistical adherence analyses were conducted. Therefore, the potential influence of treatment fidelity and adherence on outcomes cannot be determined conclusively. Furthermore, the absence of significant between-group effects likely reflects multiple factors rather than a single explanation. Possible contributing factors include limited treatment intensity within a brief outpatient group format, the exploratory adaptation of the intervention to the group setting, substantial heterogeneity of PCS symptom presentations, limited statistical power, and the possibility that the selected primary outcome measures were insufficiently sensitive to detect early psychotherapeutic changes in domains such as coping, illness adaptation, or functional recovery. Importantly, these features also reflect real-world outpatient implementation and provide a strong basis for larger trials using active controls, longer follow-up, multi-centric approaches, and outcome sets more closely aligned with functional recovery and fatigue-related disability.

## Conclusions

In this exploratory randomized controlled trial, the manual-guided group psychotherapeutic intervention did not show significant superiority over the control condition on somatic symptom burden, depressive symptoms, or anxiety symptoms. Given the limited sample size and statistical power, these findings should be interpreted cautiously. Future psychotherapeutic trials for patients with PCS should (i) focus on fatigue- and cognitive-focused components (e.g., pacing/self-management–targeted strategies) and allow targeted individual add-ons for high-burden or high-risk subgroups (e.g., premorbid anxiety/depression, multiple comorbidities, childhood trauma), (ii) prioritize functioning/participation, fatigue-related disability, health-related quality of life, illness cognitions, coping/pacing behaviour, self-efficacy as outcome measures, and (iii) pre-specify stratification by baseline severity and dominant symptom profiles (e.g., fatigue-dominant vs. cognitive impairment–prominent presentations), and consider trauma history as an additional stratifier to reduce heterogeneity-related dilution of effects.

## Data Availability

The datasets generated and/or analyzed during the current study are available from the corresponding author on reasonable request.

## Abbreviations

PCS: Post-COVID-19 condition
SSS-8: Somatic Symptom Scale-8
PHQ-9: Patient Health Questionnaire-9
GAD-7: Generalized Anxiety Disorder-7
CTS: Childhood Trauma Screener
CBT: Cognitive Behavioral Therapy
ACT: Acceptance and Commitment Therapy
TAU: Treatment as usual
IG: Intervention group
CG: Control group
